# Long-term outcome of patients with ANCA-associated vasculitis treated with plasma exchange: a retrospective, single-centre study

**DOI:** 10.1186/s13075-016-1055-5

**Published:** 2016-07-13

**Authors:** Doubravka Frausová, Zdenka Hrušková, Věra Lánská, Jana Lachmanová, Vladimír Tesař

**Affiliations:** Department of Nephrology, General University Hospital and First Faculty of Medicine, Charles University in Prague, U Nemocnice 2, 128 08 Prague 2, Czech Republic; Institute of Immunology and Microbiology, General University Hospital and First Faculty of Medicine, Charles University, Prague, Czech Republic; Statistical Unit, Institute for Clinical and Experimental Medicine, Prague, Czech Republic

**Keywords:** Alveolar haemorrhage, ANCA, Dialysis, End-stage renal disease, Outcome, Plasma exchange, Vasculitis

## Abstract

**Background:**

Plasma exchange (PLEX) has been used routinely for treatment of severe renal vasculitis and/or alveolar haemorrhage (AH) in anti-neutrophil cytoplasmic antibody (ANCA)-associated vasculitis (AAV), but the long-term benefit of PLEX in AAV remains unclear. We aimed to describe the characteristics and outcomes of patients treated with PLEX in a single centre.

**Methods:**

Patients with AAV were identified by performing a case review of medical records of 705 patients who received PLEX in a single tertiary referral centre between 2000 and 2010. Patient characteristics and outcomes were recorded. The Kaplan-Meier method, log-rank tests and Cox regression analysis were used for survival analyses.

**Results:**

A total of 94 patients with AAV were identified (44 men, 50 women; median age 60 years, range 21–90 years; 52 proteinase 3-ANCA, 41 myeloperoxidase-ANCA and 1 ANCA-negative; 8 double-positive for ANCA and anti-glomerular basement membrane; 93 newly diagnosed/1 relapse; 55 [58.5 %] required dialysis). The reasons for initiating PLEX therapy were severe renal involvement alone in 52 %, AH in 10 %, both renal involvement and AH in 35 %, and “other” in 3 %. The patients had 3–27 (median 7) PLEX sessions. At 3 months, 81 (86 %) of 94 were alive and 62 (66 %) of 94 were alive and dialysis-independent. The median follow-up was 41 months (minimum-maximum 0.5–137 months), when 56 (59.6 %) of 94 patients were alive and 47 (50 %) were dialysis-independent. The estimated overall survival rates were 75.3 % at 1 year and 61.1 % at 5 years. Patient survival decreased with increasing age at presentation (5-year survival 85 % for age <50 years, 64.4 % for ages 50–65 years, and 41 % for >65 years; *p* < 0.01 for comparison between all groups). Estimated renal survival rates were 65.5 % at 1 year and 43 % at 5 years. Renal survival was worse in patients aged >65 years than in the younger patients (5-year survival 25.1 % in patients >65 years vs. 50.8 % for those ≤65 years, *p* < 0.01). The estimated renal survival was better in patients with higher Disease Extent Index (DEI) >6 than in patients with DEI ≤6 (5-year survival 52.1 % vs. 39.4 %, *p* = 0.04), even though this was not confirmed in multivariate analysis.

**Conclusions:**

The mortality of patients presenting with severe manifestations of AAV remains high despite the use of PLEX. Older age at presentation is associated with worse overall and renal prognosis.

## Background

Anti-neutrophil cytoplasmic antibody-associated vasculitis (AAV) represents a group of autoimmune diseases, most (90–95 %) of which are associated with the presence of circulating anti-neutrophil cytoplasmic antibodies (ANCA). Three types of AAV can be distinguished: (1) microscopic polyangiitis, (2) granulomatosis with polyangiitis (formerly called *Wegener’s granulomatosis*), and (3) eosinophilic granulomatosis with polyangiitis (Churg-Strauss syndrome) [1]. Pauci-immune glomerulonephritis with various impairment of kidney function occurs in approximately 70 % of patients with AAV, and renal insufficiency constitutes a key determinant of increased AAV mortality.

ANCA likely contributes to disease pathogenesis in AAV [2], but this remains controversial. The antibodies have high molecular weight, low volume of distribution, long half-lives and low turnover rates. These factors make them a good target for therapeutic plasma exchange (PLEX) and support the use of PLEX in the treatment of AAV. The rapid removal of ANCA may result in rapid control of disease activity and prevent the accumulation of organ damage.

In the Plasma Exchange or High Dose Methyl Prednisolone as Adjunctive Therapy for Severe Renal Vasculitis (MEPEX) randomised trial, patients with severe renal vasculitis who received PLEX as adjunctive therapy had higher probability of dialysis independence at 1 year than patients treated with high-dose methylprednisolone (59 % vs. 43 % surviving dialysis-independent patients) [3]. Therapeutic PLEX has been recommended for the treatment of severe renal vasculitis (serum creatinine [S-creatinine] >500 μmol/L) [4, 5]. The benefit of PLEX in the treatment of alveolar haemorrhage (AH) or other severe organ manifestations is less clear, however. Moreover, the long-term outcomes of patients treated with PLEX in the MEPEX trial did not differ from the other arm in terms of both renal and patient survival [6]. A multicentre international randomised controlled trial, Plasma Exchange and Glucocorticoids for Treatment of Anti-Neutrophil Cytoplasm Antibody (ANCA)-Associated Vasculitis (PEXIVAS), is in progress to establish the efficacy of PLEX in reducing death and end-stage renal disease in patients with AAV with an estimated glomerular filtration rate (GFR) ≤50 ml/minute and/or AH [7]. Until the results of that trial are available, however, there is no controlled trial evidence for PLEX in the treatment of AH. In the present study, we retrospectively assessed characteristics and outcomes of patients who received PLEX for the treatment of diagnosed AAV in a single centre between 2000 and 2010.

## Methods

### Patient characteristics and clinical data

A retrospective analysis of medical records from a dialysis unit was performed for 705 consecutive patients who were treated with at least one membrane PLEX in a single tertiary referral centre between 2000 and 2010. Patients matching the definitions of AAV according to the EMA (European Medicines Agency) algorithm [8] were selected from the database. Patients who were double-positive for ANCA and anti-glomerular basement membrane (anti-GBM) with predominant anti-GBM and/or who displayed typical features of anti-GBM disease were excluded from the study. This study conformed to generally applied ethical principles and the Declaration of Helsinki. Data collection was performed within a larger project entitled Registry of ANCA-Associated Vasculitis. Informed consent was obtained from all patients, and the study was approved by the ethics committee of General University Hospital in Prague.

Data collection included baseline characteristics (vasculitis type and duration, type of ANCA, concomitant positivity of anti-GBM antibodies, organ involvement and renal function); data regarding PLEX sessions (number and type of procedures, complications); immunosuppressive treatment; outcome data (patient and renal survival) at 3, 6, 12 and 36 months and the most recent visit; and complications (infections, cardiovascular morbidity, cause of death).

Active organ involvement was retrospectively assessed with the use of the Disease Extent Index (DEI), which can range between 0 and 21 [9]. DEI was preferred to Birmingham Vasculitis Activity Score (BVAS) because of the retrospective data collection and lower risk of misscoring with DEI. Severe renal involvement was defined as the need for haemodialysis or S-creatinine above 500 μmol/L, or as GFR below 20 ml/minute together with either clinical evidence of rapid progression or severe finding in the renal biopsy. AH was defined as the presence of diffuse pulmonary infiltrates on a chest x-ray or computed tomographic scan and either observed haemoptysis or unexplained anaemia (<100 g/L)/documented drop in haemoglobin (>10 g/L) from <100 g/L.

### Immunosuppressive treatment

All patients were treated with combined immunosuppressive treatment consisting of corticosteroids (typically started with three pulses of intravenous methylprednisolone followed by oral prednisone) and cyclophosphamide (CYC) given as an oral daily dose of 2 mg/kg/day (preferred until 2002) or (since 2003) as intravenous pulses of 15 mg/kg/pulse every 2–3 weeks (with dose reduction based on age and renal function) [10]. CYC was administered for 3–6 months, and then the patients were switched to azathioprine, typically used for 2 years. For patients using allopurinol or patients intolerant of azathioprine, mycophenolate mofetil was used as the maintenance agent. Prophylaxis of *Pneumocystis jirovecii* with co-trimoxazole was routinely used.

### Plasma exchange

PLEX was used as an adjunctive therapy added to standard immunosuppression. In this study, PLEX was performed by using a filter separation technique in all patients. The most common PLEX dose included seven PLEX sessions performed within 14 days (based on the regimen used in the MEPEX trial [3]), with possible dose reduction or prolongation according to the patient’s clinical status. The exchanged volume was calculated using a nomogram (with calculation based on gender, body height and weight, and haematocrit) and corresponded to one plasma volume per one session. A central venous catheter (11.5-French), preferably inserted into the right internal jugular vein, was used as vascular access in all patients. Standard anti-coagulation was provided by administering heparin; the initial dose was 35 IU per kilogram of body weight, and the dose of about 10–20 IU/kg body weight/h was administered during each session, adapted according to the patient’s partial thromboplastin time.

Until 2004, fresh frozen plasma was preferably used as the replacement solution. Since then, 5 % albumin has been used routinely, and fresh frozen plasma is used only in patients with severe active bleeding (e.g., pulmonary haemorrhage). Blood pressure, body temperature and heart rate were regularly monitored during each procedure. The frequency of monitoring of coagulation parameters, blood count, ANCA and total immunoglobulin levels differed with respect to each patient’s status.

### Statistical analysis

Continuous variables are presented as mean ± SD if normally distributed or as median (minimum-maximum) if skewed. Kaplan-Meier survival curves, log-rank tests and Cox regression analysis were used to assess survival. A two-sided *p* value <0.05 was considered statistically significant.

## Results

### Patient characteristics

A total of 94 patients with AAV were identified. Basic patient characteristics are displayed in Table 1. All but one patient received PLEX at the time of diagnosis, and one patient was treated at relapse. Of 94 patients, 8 were double-positive for ANCA and anti-GBM; four these were proteinase 3 (PR3)-ANCA-positive and four were myeloperoxidase (MPO)-ANCA-positive. The most common reason for PLEX therapy was severe renal involvement (either alone or in combination with AH), which was present in 87 % of patients. Active renal involvement was present in 97 % patients; lung involvement in 65 %; and ear, nose and throat involvement in 27 %. The patients received 3–27 PLEX sessions (median 7, interquartile range 6–9).

**Table 1:** Baseline patient characteristics

Patient characteristics (*n* = 94)	Data
Male gender, *n* (%)	44 (47 %)
Age, years, median (min-max)	60 (21–90)
ANCA type	
PR3, *n* (%)	47 (50 %)
MPO, *n* (%)	37 (39 %)
Negative, *n* (%)	1 (1 %)
Double-positive for ANCA and anti-GBM, *n* (%)	8 (9 %)
Reason for PLEX	
Severe renal involvement without AH, *n* (%)	49 (52 %)
AH without severe renal involvement, *n* (%)	9 (10 %)
Both severe renal involvement and AH, *n* (%)	33 (35 %)
Other, *n* (%)	3 (3 %)
Serum creatinine in non-dialysed, median (min-max)	369 (40–693)
Patients on dialysis, *n* (%)	55 (59 %)
Active organ involvement	
Kidney, *n* (%)	91 (97 %)
Lungs, *n* (%)	61 (65 %)
ENT, *n* (%)	25 (27 %)
Eyes, *n* (%)	5 (5 %)
Skin, *n* (%)	9 (10 %)
Peripheral nervous system, *n* (%)	7 (7 %)
Other, *n* (%)	3 (3 %)

*Abbreviations: AH* alveolar haemorrhage, *ANCA* anti-neutrophil cytoplasm antibodies, *ENT* ear, nose and throat, *GBM* glomerular basement membrane, *max* maximum, *min* minimum, *MPO* myeloperoxidase, *PLEX* plasma exchange, *PR3* proteinase 3

### Outcomes

The median time of follow-up was 41 months (min-max 0.5–137 months), when 56 (59.6 %) of 94 patients were alive and 47 (50 %) were dialysis-independent. At 3 months, 81 (86 %) of 94 were alive and 62 (66 %) of 94 were alive and dialysis-independent (Table 2).

**Table 2:** Outcome data for patient and renal survival

Absolute time since PLEX	Surviving, *n* (%)	RRT in survivors, *n* (%)
0 months (*n* = 94)	94 (100 %)	55 (58.5 %)
3 months (*n* = 94)	81 (86.2 %)	19 (23.5 %)
1 year (*n* = 94)	68 (72.3 %)	14 (20.6 %)
3 years (*n* = 79)	49 (62 %)	9 (18.4 %)
5 years (*n* = 61)	26 (42.6 %)	5 (19.2 %)
End of follow-up (*n* = 94)	56 (59.6 %)	9 (16.1 %)

*PLEX* plasma exchange, *RRT* renal replacement therapy

#### Overall survival

**Fig. 1:**
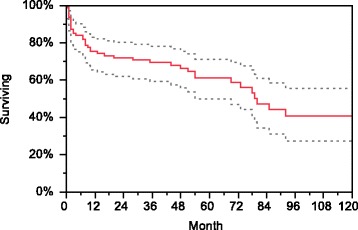
Estimated overall patient survival (*dotted lines* represent 95 % CI)

**Fig. 2:**
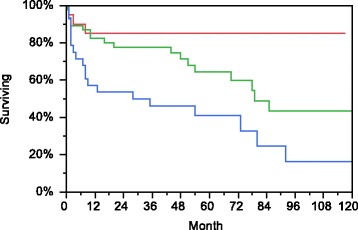
Significant difference in estimated overall patient survival in different age groups (*red line* <50 years, *green line* 50–65 years and *blue line* >65 years)

The estimated overall survival rates were 75.3 % at 1 year and 61.1 % at 5 years (Fig. 1). The estimated 5-year survival rates were better in younger patients than in the older ones (85 % in patients <50 years, 64.4 % in patients aged 50–65 years and 41 % in patients >65 years; *p* < 0.01 for comparison between all groups) (Fig. 2). Age remained also the only significant risk factor for death in both univariate and multivariate Cox regression models (relative risk [RR] 2.9, 95 % CI 1.5–5.8, *p* < 0.01 when age groups ≤65 and >65 years were used in the model). No difference in overall survival was found between patients with S-creatinine below or above 500 μmol/L. Survival did not differ in the groups with different antibodies (PR3 vs. MPO vs. double-positive anti-GBM and ANCA) or between patients with vs. without AH (detailed data not shown). We also did not find any difference in overall survival between patients diagnosed in the 2000–2005 period vs. 2006–2010.

#### Renal survival

**Fig. 3:**
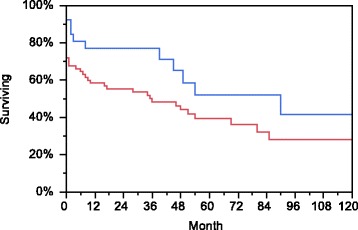
Difference in estimated renal survival in patients with Disease Extent Index (DEI) ≤6 (*red line*) vs. DEI >6 (*blue line*)

Estimated renal survival rates at 1 year were 65.5 % and 43 % at 5 years. Renal survival was worse in patients older than 65 years of age than in the younger ones (5-year survival 25.1 % in patients >65 years old vs. 50.8 % in patients aged ≤65 years, *p* < 0.01). No difference in renal survival was found in subgroups with different S-creatinine levels below or above 500 μmol/L. The renal survival did not differ in the groups with different antibodies (PR3 vs. MPO vs. double anti-GBM and ANCA). Using Kaplan-Meier analysis, no difference in renal survival was found between patients with and without AH, however, the renal survival was better in patients with higher DEI (above 6) than in patients with DEI ≤ 6 (5-year survival 52.1 % vs. 39.4 %, *p* = 0.04) (Fig. 3). More patients with lower DEI required dialysis at entry (45 of 68) than among the group of patients with a higher DEI (10 of 26, *p* = 0.02). In the multivariate analysis, the DEI was not confirmed as an independent risk factor for renal death.

#### Causes of death

The leading cause of death was infection (observed in 18 patients), followed by cardiovascular causes (7 patients). In ten patients, the cause of death was not known or was unavailable. The remaining causes of death were observed in individuals only (one malignancy, one liver failure, one haemorrhagic shock).

## Discussion

PLEX has been used routinely in the treatment of severe manifestations of ANCA-associated vasculitides, but evidence from randomised trials supporting its long-term benefit is missing. Moreover, the situation in randomised clinical trials is always artificial, and there is a bias towards the selection of “healthier” and/or “more compliant” patients. Thus, the assessment of outcomes in a real situation in clinical practice is very important and provides information on the routine use of PLEX in patients with AAV. In this study, we included a large cohort of patients with AAV treated with PLEX in a single centre, and the results show that the mortality of patients presenting with severe manifestations of AAV remains high despite the use of PLEX.

Even though direct comparison is difficult because the patients’ characteristics differed, our results regarding long-term patient survival were generally similar to or slightly better than those described in the long-term follow-up of the MEPEX trial (median follow-up 3.41 vs. 3.95 years, during which time there were 38 (40.4 %) of 94 and 35 (50.7 %) of 69 deaths in our study and the MEPEX trial, respectively [6]). The important differences between our study and the MEPEX trial include the use of intravenous CYC in the majority of our patients, while only oral CYC was used in the older MEPEX trial; in addition, we enrolled patients with (sometimes severe) AH in our study. The lower cumulative dose of CYC achieved with its intravenous use may have contributed to better patient outcomes. This was also demonstrated in another retrospective study by Pepper et al. on the use of PLEX and intravenous CYC, in which only patients requiring dialysis were included [11]. Our patients’ 1-year survival rate was lower than that in the study by Pepper et al, but their study period (2005–2010) and patients’ characteristics (percentage of patients with AH, percentage of ANCA subtypes) differed. Interestingly, we did not find any difference in the survival of patients with vs. without AH, although the patients with AH were similarly distributed among those dialysed and those not dialysed (data not shown). While the overall mortality was associated with the degree of renal insufficiency in a previous retrospective study on AH [12], in our study no difference in survival between patients requiring vs. not requiring dialysis at presentation was found, and the only factor clearly negatively influencing prognosis in both studies was increasing age.

In our centre, during the whole study period, PLEX was routinely used for (almost) all patients presenting with renal failure and the vast majority of patients presenting with AH. Thus, we were not able to use a matched control group of patients not treated with PLEX, as this would certainly have been subject to bias. While researchers in a recent single-centre study found no significant difference between groups with vs. without PLEX [13], the authors of a meta-analysis of previously published studies [14] concluded that PLEX in either renal vasculitis or idiopathic rapidly progressive glomerulonephritis may decrease the composite end point of end-stage renal disease or death, but the effect of PLEX for death alone was not significant (RR 1.01, 95 % CI 0.71–1.4, *p* = 0.9), and the authors admitted having insufficient statistical information, given the relative lack of data.

The renal survival results in our study were also similar to previously published data [6, 11]. In some studies, researchers have found that the efficacy of PLEX may be related to ANCA specificity (advantageous effect observed in PR3-ANCA-positive patients) [15], while other studies did not confirm that observation [16], in accordance with our results, even though we did not use PLEX in patients with better-preserved renal function. Interestingly, we found that higher DEI (influenced by extrarenal involvement) tended to be associated with better renal survival, suggesting that extrarenal symptoms may enable earlier diagnosis, which is probably crucial for favourable long-term outcomes of patients with AAV. In line with this hypothesis, the number of patients dialysed at entry was less in patients with higher DEI in this study, even though this observation is limited by the fact that the role of DEI as an independent predictor of end-stage renal disease was not confirmed in the multivariate analysis.

The safety of PLEX remains an important issue. In this study, we did not observe any death or major adverse events directly related to PLEX. In theory, PLEX may lead to removal of coagulation factors and to worsening of AH. Nevertheless, this may be prevented by a substitution of fresh frozen plasma at the end of PLEX [17]. In our study, infection, and not active vasculitis, was the most common cause of death, which is consistent with previous reports [18], suggesting that our current treatment strategies are efficacious but may be too aggressive in some patients. Surprisingly, even in patients treated with rituximab in a randomised trial [19], the rate of adverse events was not lower than in CYC/azathioprine-treated patients, which implies that high-dose corticosteroids may also play an important role in treatment toxicity.

On one hand, we are aware of possible limitations of this study, caused mainly by its retrospective nature and the lack of a control group, as discussed above. On the other hand, we present data on a relatively large population of patients from a single centre that largely confirm the results of previous studies. The use of the DEI instead of the more routinely used BVAS might have influenced the assessment of activity and its potential relationship; however, especially in patients diagnosed a long time ago, the retrospective BVAS assessment may not be valid. In patients with suspected AH, we always tried to exclude infection or other causes of bleeding, but it possible that misclassification might have occurred in some patients.

## Conclusions

Our results show that mortality of patients presenting with severe manifestations of AAV remains high despite the use of PLEX. Older age at presentation is associated with worse prognosis. Higher DEI with extrarenal involvement may enable earlier diagnosis and improve renal survival, even though the independent predictive value of DEI has not been confirmed in our multivariate analysis.

The prognosis of AAV seems to have been improving in recent decades [20], including the severe manifestations [12], and it is currently unclear to what extent PLEX contributes to this process [17]. Hopefully, the ongoing PEXIVAS trial will shed more light on the indications for PLEX in patients with AAV, particularly the role of PLEX in patients with non-dialysis-dependent renal failure and in patients with AH.

## Abbreviations

AAV, anti-neutrophil cytoplasmic antibody-associated vasculitis; AH, alveolar haemorrhage; ANCA, anti-neutrophil cytoplasmic antibodies; BVAS, Birmingham Vasculitis Activity Score; CYC, cyclophosphamide; DEI, Disease Extent Index; ENT, ear, nose and throat; GBM, glomerular basement membrane; GFR, glomerular filtration rate; MEPEX, Plasma Exchange or High Dose Methyl Prednisolone as Adjunctive Therapy for Severe Renal Vasculitis randomised trial; MPO, myeloperoxidase; PEXIVAS, Plasma Exchange and Glucocorticoids for Treatment of Anti-Neutrophil Cytoplasm Antibody (ANCA)-Associated Vasculitis randomised controlled trial; PLEX, plasma exchange; PR3, proteinase 3; RR, relative risk; RRT, renal replacement therapy; S-creatinine, serum creatinine
